# State of the Art on Biomaterials for Soft Tissue Augmentation in the Oral Cavity. Part I: Natural Polymers-Based Biomaterials

**DOI:** 10.3390/polym12081850

**Published:** 2020-08-18

**Authors:** Manuel Toledano, Manuel Toledano-Osorio, Álvaro Carrasco-Carmona, Cristina Vallecillo, Christopher D. Lynch, María T. Osorio, Raquel Osorio

**Affiliations:** 1 Dental Materials Section, Faculty of Dentistry, University of Granada, Colegio Máximo de Cartuja s/n, 18071 Granada, Spain; toledano@ugr.es (M.T.); alcarcar94@correo.ugr.es (Á.C.-C.); cvallecillorivas@hotmail.com (C.V.); mtoleosorio@gmail.com (M.T.O.); rosorio@ugr.es (R.O.); 2 Restorative Dentistry, University Dental School & Hospital, University College Cork, Wilton, Cork T12 E8YV, Ireland

**Keywords:** soft tissue, augmentation, graft, oral, matrix, tissue engineering, scaffolds

## Abstract

Oral soft tissue thickening or grafting procedures are often necessary to cover tooth recession, re-establish an adequate width of keratinized tissue, correct mucogingival deformities improving esthetics, prepare a site for an implant or prosthetics, for ridge preservation procedures, and soft tissue contouring around dental implants. Gingival recession and root or implant exposure are commonly associated and have led to mucogingival deficiencies that have traditionally been treated with free gingival grafts and autogenous soft tissue grafts. The latter represents the gold standard in acquiring a functionally adequate zone of keratinized attached gingiva. However, soft tissue substitutes are more usually employed because they lessen morbidity and abbreviate surgical time. This review is aimed at assessing oral soft tissue augmentation techniques and biomaterials used from existing literature, principally concerning scaffolds from both human and animal-based tissue derivatives matrices. In order to avoid the use of human donor tissue, the xenogenic collagen matrices are proposed for soft tissue augmentation. In general, all of them have provided the remodeling processes and enhanced the formation of new connective tissue within the matrix body.

## 1. Introduction to Soft Tissue Augmentation. Background of the Techniques Used to Gain Soft Tissue Width and Volume

Nowadays, in implantology and periodontics, the interest in the management and care of soft tissues is raising. In oral cavities of some patients, it has been observed that gingival recessions, trauma, infections, and tumors can produce a lack of soft tissue amount. This can lead to a need for a soft tissue reconstruction [1]. Polymers are crucial biomaterials for this purpose, and, according to their origin, they can be classified as natural polymers (including xenogenic-derived collagen), synthetic polymer materials (like poly(lactic acid)) or polymer composites. This last group is defined as the combination of two or more different materials for the obtention of specific physical, chemical or mechanical properties. Natural polymers possess great biodegradability, biocompatibility and safety, and exhibit several advantages when compared to synthetic ones, such as their natural remodeling, the susceptibility to proteolytic degradation triggered by cells or the ability to present receptor-binding ligands to cells [2]. On the other hand, synthetic polymers hold the advantage of having a well-controlled and reproducible molecular structure, modifying its mechanical properties depending on the needs [3].

### 1.1. Former Treatments for Gingival Recession, Root or Implant Exposure and Soft Tissue Thickening

**Figure 1:**
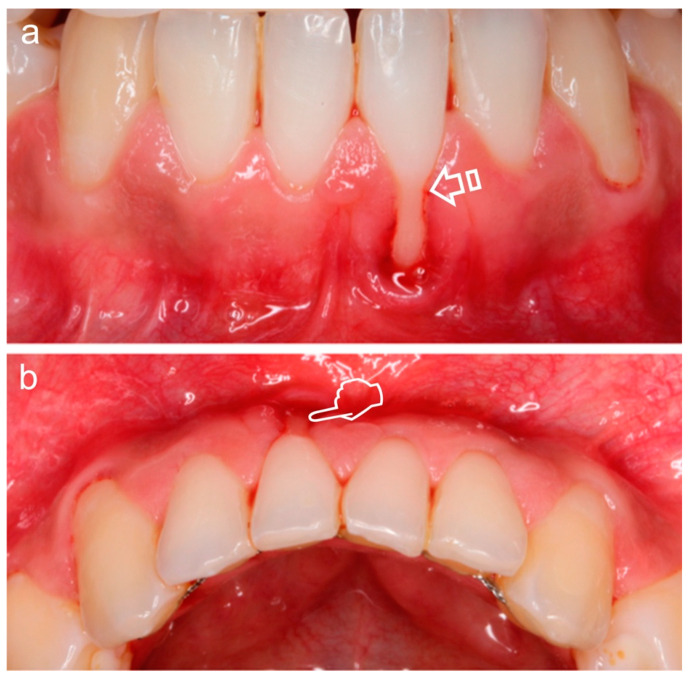
Miller Class II Gingival Recession Defect [7]. The gingival margin is located apical to the cementoenamel junction (CEJ) (arrow), resulting in exposure of the root surface. Frontal (**a**) and occlusal (**b**) vision. The loss of volume (pointer) is better appreciated in (**b**).

**Figure 2:**
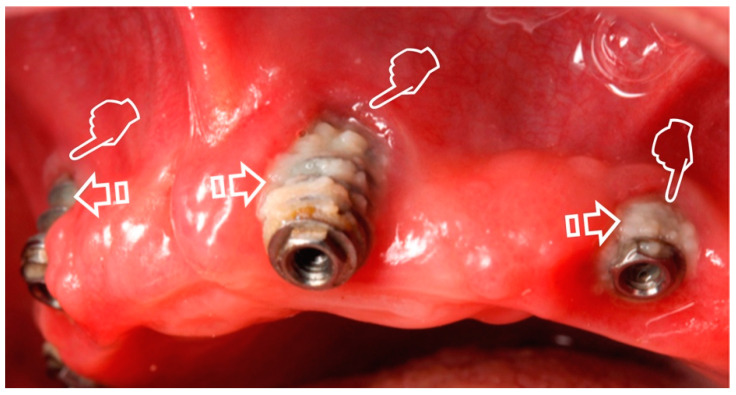
Gingival recession (pointers) caused by peri-implantitis with plaque deposits (arrows) on the implant surface.

Gingival recession and subsequent root surface (Figure 1) or implant exposure (Figure 2) are commonly associated [4]. The aesthetic concern is normally present in these clinical circumstances, where treatment of mucogingival deficiencies has become a large part of these practices. Hence, soft tissue augmentation procedures are often required around teeth or implants, in modern dental treatments. These surgeries are categorized into two main groups: methods involved in keratinized mucosa generation and/or widening, and those affecting the volume/thickness increasing of soft tissue. When considering the long-term stability of the tissue and esthetics, a very important factor is the mucosal thickness. Soft and hard tissue stability and dental implants long-term preservation are increased when a wider keratinized mucosa zone is present. Furthermore, a lack of this mucosa is related to an oral hygiene deficiency and a bigger soft tissue recession. It is therefore clinically recommended a keratinized mucosa width of 2 mm, which is the same size that it is suggested for an adequate keratinized gingiva zone around the teeth [5]. When the mucosa thickness is higher than 2 mm, the human eye is no longer able to detect light reflection (translucency) differences of zirconia or titanium abutments underneath the soft tissue [6].

As stated above, the covering of a recession and the preparation of a concrete size for an implant or prosthetic, contouring of soft tissue around dental implants and procedures for ridge preservation often require soft tissue thickening [8]. In the last 50 years, soft tissue grafting has been a viable alternative for the augmentation of tissue thickness, the generation of adequate keratinized tissue volumes, the correction of mucogingival deformities and esthetics improvement at dental implant sites [9]. The number of these procedures has increased in recent years along with dental implant therapy for a wide number of indications [10]. Clinical trials have shown that in order to offset the around 30% of shrinkage of the soft tissue grafting during the healing process, it is necessary to harvest a graft with a higher width than the defect that requires soft tissue augmentation. This can result in complications at the donor site and postoperative discomfort [9]. At the moment, randomized controlled clinical trials, including those centered in soft tissue volume gain, are lacking. In contrast, there are some of these studies available in keratinized tissue augmentations but, as they are sponsored by certain companies, a higher bias risk does exist. Therefore, in order to increase the benefit for patients and clinicians and to obtain data on optimal materials, more independent and self-funded studies are required [5].

### 1.2. Periodontal Plastic Surgery and Its Applicability. Autogenous Soft Tissue Graft

Periodontal plastic surgery can be defined as the compilation of surgical procedures undertaken to avoid or correct traumatic, anatomic, disease or development-caused defects to the alveolar mucosa, gingiva or bone [11]. Like any other tissue, the basic components of tissue engineering approach (cells, growth factors and scaffold or extracellular matrix) will be required for periodontal tissue regeneration [12]. These procedures have been proposed to treat mucogingival anomalies and achieve both functional and aesthetic outcomes, increasing survival rates of teeth and implants [13]. Primary clinical indications include keratinized tissue gain, augmentation of soft tissue volume and recession coverage. In order to increase missing or lost keratinized tissue, the free gingival graft (FGG) was introduced [9]. FGG, as an example of autogenous soft tissue, has been proposed but the lack of predictability in achieving total root coverage and the compromised blood supply must also be considered as drawbacks of this technique. Other shortcomings have been described when the outcomes of FGG were analyzed, including incorrect recipient site preparation, inadequate recipient bed adaptation, failure for the graft stabilization and an inadequate graft size and thickness [1].

**Figure 3:**
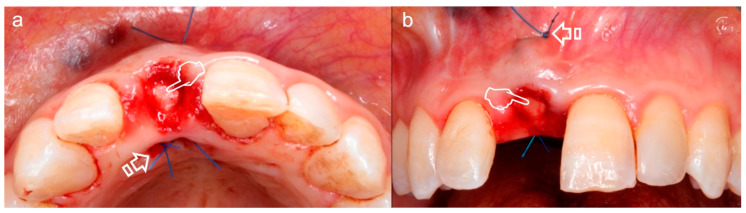
Subepithelial connective tissue graft for volume gain after immediate implant placement. The gain of the volume is better appreciated in (**a**) where an augmentation of the phenotype thickness is shown. A portion of the graft can be observed at the surgical area (pointers) (**a**,**b**). The sutures anchorage the graft (arrows) (**a**,**b**).

**Figure 4:**
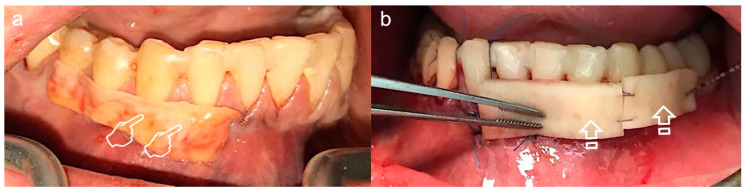
Split-mouth recessions treatment, connective tissue graft (CTG) for quadrant IV (**a**) and acellular dermal matrix (ADM) for quadrant III (**b**). Try-on of the harvested CTG (pointers) and ADM (arrows) for decision-making: large enough for a tunnel procedure.

For root or peri-implant coverage care, the increase of the width of attached gingiva and the increase of the keratinized tissue, the most predictable clinical procedure is the subepithelial connective tissue graft (CTG) [4] (Figure 3 and Figure 4a). With extensive evidence, CTG has been defined as the preferred choice for the treatment of gingival/mucosal recessions at defect sites and teeth, thickness augment of soft tissue, hiding visible components of implants and discolored roots and reconstruction of interdental papilla [5,9]. Zucchelli et al. [9] suggested a biologic filler performance for the CTG as, during early wound repairment, it is able to improve flap stability and adaptation to the root. This results in an augmentation of the gingival phenotype thickness and the potential to achieve complete root coverage. A suitable biomaterial requires two properties in order to be applied for soft tissue volume increase: (i) to exhibit volume stability over a period of time, and (ii) a satisfactory biological behavior for modeling and remodeling procedures [14].

**Figure 5:**
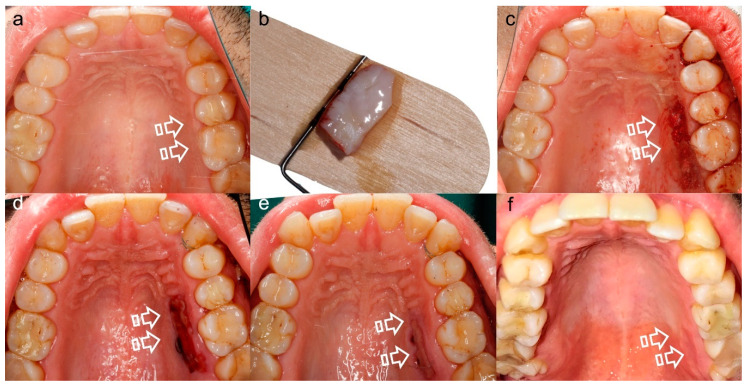
Palatal Wound Healing. (**a**) Prior to surgery, (**b**) harvested connective tissue graft, (**c**) post-surgery, (**d**) 7 days follow-up, (**e**) 15 days follow-up, (**f**) 21 days follow-up. This sequence of images shows the morbidity associated with the donor tissue site (arrows), that besides is scarce to supply multiple recession defects treatment.

Nevertheless, patient morbidity associated with the second surgical site, surgical time augmentation, the donor tissue scarce supply for multiple recession defects treatment and subsequent healing pose a crucial drawback [4,5,14,15] (Figure 5). Buccal fat pad and free buccal mucosa grafts have also been proposed [1]. Thereby, an alternative treatment for the autogenous soft tissue graft is recommended based on the good clinical integration of collagen-based dermal matrices in implant and plastic periodontal surgery [6].

## 2. Scaffolds for Soft Tissue Augmentation

Some of the major areas in biomaterial research are the design and fabrication of scaffolds. These are also major themes for the engineering of tissues and research on regenerative medicine. In detail, scaffolds are needed to regenerate tissues or restore function, acting as a temporary matrix when they are employed in order to secure the proliferation of cells and the deposition of the extracellular matrix. They allow the subsequent ingrowth until the complete restoration or regeneration of the tissue is achieved [16]. Scaffolds can be defined as biomaterials with a three-dimensional porous solid structure. These biomaterials are synthesized with the objective of undertaking some or all of the following functions: (i) the promotion of interactions between cell and biomaterials, adhesion of cells and the deposition of an extracellular matrix (ECM), (ii) to allow suitable gases, nutrients and regulatory transport that enables the survival, differentiation and proliferation of cells, (iii) a controllable biodegradation rate similar to that of tissue regeneration under specific culture conditions, and (iv) to trigger a minimal toxicity or inflammation degree in vivo [16].

### 2.1. Human-Based Tissue Derivatives for Soft Tissue Grafts

Within the group of natural and cadaveric scaffolds, the acellular dermal matrix (ADM) is one of the most representative soft tissue grafts with human skin origin that is able to bear a decellularization process [17,18]. It can act as a scaffold to promote revascularization and the migration of cells from adjacent host tissues. Based on the results of preclinical studies, ADMs have been proposed as suitable scaffolds for the three-dimensional thickening of soft tissue, as they exhibit good biocompatibility and biodegrading features [6]. Furthermore, ADM preserves space for new periodontal tissue formation, which is paramount for successful periodontal tissue regeneration [1] (Figure 4b). Recently, ADM has been employed in procedures for soft tissue augmentation and root coverage at implant or tooth places. Specifically, ADM is used when there is a need to avoid the generation of a second surgical site and to minimize the morbidity in the patient. This barrier is more susceptible to shrinkage, which explains the reduction of tissue thickness that has been reported, which looks like a “scar” [15]. A significant gingival margin relapse in several ADM-treated gingiva recessions has also been described. It has been attributed to a potential inability of ADM to induce overlying epithelia keratinization [15]. Nevertheless, keratinocyte and/or fibroblast incorporation on acellular scaffolds have been demonstrated to be well tolerated by hosts and it also enhances the formation of blood vessels and migration of cells by secreting specific growth factors. Even more, the seeding of fibroblasts without keratinocytes has shown that it does not affect gingival epithelium keratinization [19]. Due to its color matching, biocompatibility and horizontal gain, allogenic ADM has been defined as a viable alternative for the treatment of ridge deformities in soft tissue. However, such tissue gain has only been achieved in a few certain cases [6].

Human amniotic membranes (HAM) are comprised by three main components: (i) a single layer of epithelium, (ii) a thick basement membrane, and (iii) an avascular layer of collagen. Numerous growth factors are enclosed in the avascular stroma. Among them are included the epidermal growth factor, transforming growth factors alpha and beta (TGF-*α*, TGF-*β*), fibroblast growth factor-2, and the keratinocyte growth factor [20]. All of them contribute to immunomodulatory, anti-inflammatory, antiviral, antimicrobial, analgesic and anti-scarring properties. HAM also encourages angiogenesis, collagen deposition and epithelial wound healing. However, this allograft poses some drawbacks that include handling difficulty and fast degradation [15]. Nevertheless, using human-based tissue derivatives may be linked to ethical concerns and the potential risk of disease transmission [4].

### 2.2. Animal-Based Tissue Derivatives for Soft Tissue Remodeling

The use of human donor tissue has been avoided by employing porcine instead of cadaveric dermal matrices. They offer multiple advantages, including higher availability, cost reduction or the ability to be harvested in abundance. The group of xenogenic collagen matrices comprises a set of current collagen membranes, all of them aimed to allow the process of remodeling and the enhancement of new connective tissue formation in the matrix body [14]. These collagen matrices (mainly types I and III) exhibit superior properties, including its biodegradation without eliciting foreign-body reactions, fast vascularization, good tissue integration, hemostatic properties, chemotactic action for fibroblasts, osteoblastic adhesion, weak immunogenicity and its biocompatibility and ability to promote wound healing. They can be reabsorbed by the action of collagenases/proteases enzymatic degradation, bacterial proteases and enzymes derived from macrophages/polymorphonuclear leukocytes [2].

**Figure 6:**
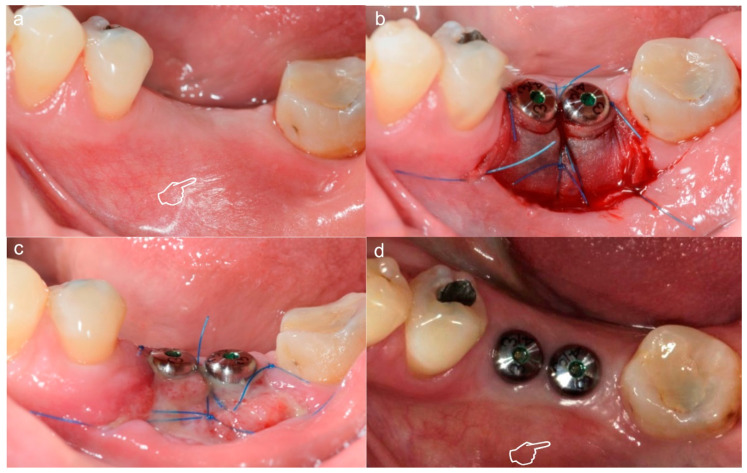
Soft tissue augmentation in an implants’ second stage surgery using Mucograft. (**a**) Prior surgery (**b**) Post-surgery with the matrix fixed to the recipient bed (**c**) 2 weeks follow-up (**d**) 3 months follow-up. This matrix was used as contouring of soft tissue around dental implants. The reduced ridge (pointer in (**a**)) was thickened after soft tissue augmentation (pointer in (**d**)).

Mucograft (Geistlich Pharma AG, Wolhusen, Switzerland) is a non-cross-linked, resorbable, porcine bilayered type I and III collagen matrix. It exhibits an occlusive compacted collagen layer with a smooth texture that promotes cell adhesion. The porous structure that faces the host tissue enables angiogenesis and tissue integration. The porosity of scaffolds (membranes and matrices) facilitates the angiogenesis [21]. In the construction of a successful engineered tissue, vascularization plays a key role. Oxygen received though diffusion is only received by cells that are located in a 100–200 µm radius from blood vessels [22]. It is possible for this matrix to increase the width of keratinized tissue, but a lack of the cellular components required for such formation has raised several concerns about its potential [23]. Notwithstanding, mucograft has been proposed as an alternative scaffold for fibroblast and round-shaped [24] keratinocytes proliferation [15]. The secretion of growth factors and other proteins, such as fibronectin, cytokines, glycosaminoglycans and human dermal collagen by fibroblasts, after placing mucograft, can lead to a metabolically active matrix structure. This assembly can act as a promoter of adjacent cells colonization of the defect wound, re-epithelialization and angiogenesis. It can also encourage keratinocytes migration and attachment while also serving as an active agent for wound healing (Figure 6). In addition, after the implantation of these tissue-engineered constructs, such as mucograft, there is a production of angiogenic-related biomarkers, including PDGF-BB, angiostatin, angiogenin, VEGF, interleukin (IL)-8, FGF-2, granulocyte-macrophage colony-stimulating factor (GM-CSF), tissue inhibitor of metalloproteinase (TIMP)-1, TIMP-2 and interferon gamma-induced protein 10 [19].

**Figure 7:**
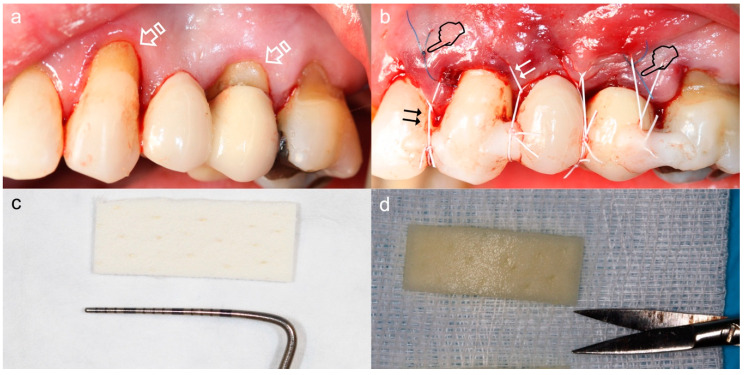
Coronally advanced tunnel technique for management of gingival recession using Mucoderm. (**a**) Situation prior to surgery, recessions and non-carious cervical lesions (arrows). (**b**) Situation post-surgery. The graft was inserted into the tunnel using a mattress stitch (pointers) and the entire complex (tunnel and injector) was coronally repositioned using suspension stitches (double arrows) anchored to the retention of composite placed at the contact points. (**c**) Matrix before hydration. Membrane measurements have been verified to be adequate for the reception area. (**d**) Matrix after hydration in a sterile saline solution for ten minutes.

Mucoderm (botiss gmbh, Berlin, Germany) is a porcine-derived acellular dermal matrix (PADM) obtained by eliminating the antigenic constituents using a multi-step procedure, which enhances the preservation of the whole extracellular collagenous matrix [4]. This matrix can serve as a three-dimensional scaffold to facilitate fibroblasts and endothelial cells proliferation, leading to fast revascularization [25] (Figure 7).

**Figure 8:**
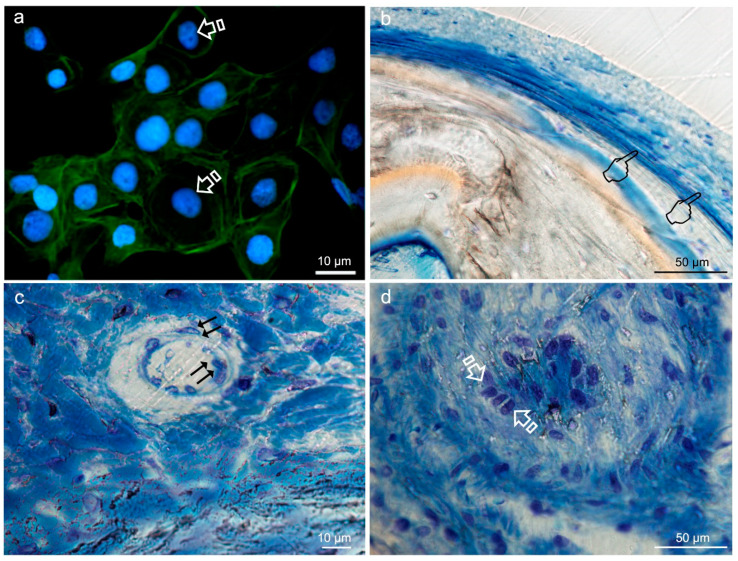
Typical round-shaped images of the keratinocytes cytoskeleton (arrows) obtained by immunofluorescence, from Niculiţe et al. [26]. Copyright MDPI, 2018 (**a**). Bone histology obtained after using silica-loaded carboxilated doxycycline-doped membranes (**b**,**c**) and blank control (no membrane) (**d**), by coloration with toluidine blue to visualize the elongated fibroblasts (pointers in (**b**)), fusiform endothelial cells of blood vessels (double arrows in **c**) and cuboid osteoblastic cells (faced arrows in (**d**)), at 6 weeks of healing time in animal experimentation (Toledano et al./MAT2017-85999P).

The 3D matrices surface characteristics, such as porosity and interconnectivity, influence the matrices performance. PADM matrices also act as scaffolds to allow human gingival fibroblasts, osteoblasts, endothelial and oral keratinocytes in-growth and repopulation (Figure 8). Its integration into the surrounding tissues will essentially depend on the infiltration of all these cells and blood vessels [4]. Besides, the matrix must be well-tolerated without eliciting a foreign body or immunological response. Mucoderm acts, specifically, as a substrate for the attachment, growth and differentiation of keratinocytes. This can be caused by the similarity to the normal debris of the matrix biological structure. Extracellular matrix homeostasis is regulated by dermal fibroblasts. This is vital for the growth and differentiation of keratinocytes, which form the external epithelial layer and provide the barrier effect. Both fibroblasts and keratinocytes communicate via cross-talk, paracrine-signaling that is crucial for the soft tissue healing. In fact, cytokines and growth factors expression, such as fibroblast growth factor (FGF)-2, platelet-derived growth factor (PDGF), bone morphogenetic proteins (BMPs), insulin-like growth factor-1 and VEGF, are different from other tissue-engineered constructs based on only one cell type. This suggests an active role for both fibroblasts and keratinocytes in the reproduction of fully-developed epithelium [19]. In order to ensure implanted matrix perfusion, microvessels ingrowth is essential. This causes sufficient matrix incorporation that prevents avascular wound infections and warrants high-quality wound healing. New capillaries formation requires endothelial cells activation, proliferation and migration from preexisting vessels through angiogenesis [4]. Mucoderm has been proposed as an enamel matrix derivative carrier in gingival recessions treatment [15].

**Figure 9:**
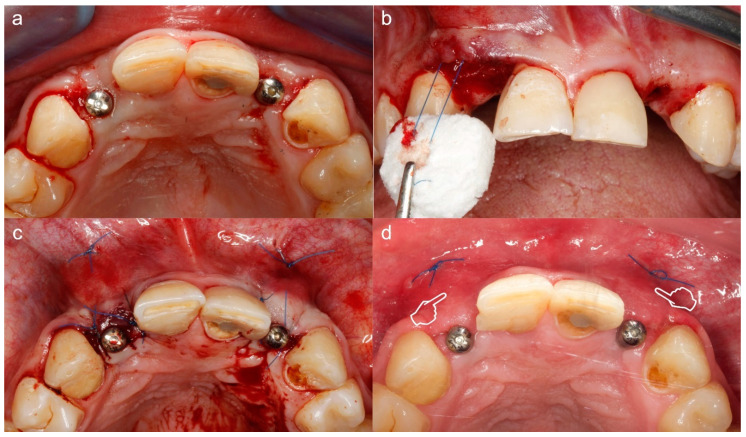
Soft tissue augmentation around implants using Fibro-gide. (**a**) Situation prior to surgery. (**b**) Matrix placement using a mattress stitch to pull the matrix through the full thickness envelope flap. (**c**) Post-surgery situation. (**d**) The 14 days follow-up. The soft tissue augmentation effect was clinically evidenced in (**d**) (pointers).

The main advantage of Fibro-gide (Geistlich Pharma AG, Wolhusen, Switzerland) is its capacity to warrant good tissue volume stability. This collagen matrix undertakes a cross-linking process that also provides some elasticity. Therefore, the mechanical and biodegradable stabilities have been reinforced by using different physical, biological and chemical cross-linking methods to obtain, generally, cross-linked collagen. This allowed the obtention of enhanced tensile strength and the prolongation of collagen degradation time. However, collagen implants degradation can produce residues or secondary products with toxic effects, hindering their application. Furthermore, some polysaccharides have exhibited a success degree for the cross-linking of collagen membranes. Physical treatments, such as ultraviolet, gamma or microwave irradiation or dehydrothermal and heat treatment, and biological methods (e.g., transglutaminase) can be applied as an alternative for the introduction of efficient cross-linking. Nevertheless, these are not the only problems with cross-linked collagen, as it has been observed that cross-linked membranes exhibit longer integrity of the membrane with surrounding blood vessels and tissues when compared with non-cross-linked ones. These enzymatically and chemically cross-linked collagen matrices have demonstrated a delay in experimental animals’ angiogenesis [2]. In addition, fibro-gide possesses a porous layer that favors fibroblasts ingrowth, tissue integration, matrix biosynthesis and angiogenesis [15]. It also requires submerged healing (Figure 9).

DynaMatrix (Keystone Dental, Burlington, MA, USA), which is an extracellular matrix, is used to provide a viable scaffold for fibroblasts, epithelium from adjacent tissue and blood vessel proliferation. Due to its high elasticity, this matrix is able to regain its original volume within the first few minutes after application [14]. Nevertheless, some clinical considerations concerning their clinical stabilization and characteristics in comparison with connective tissue and free gingival grafts are scarce in the bibliography, at the moment, and its direct application must be done with caution [15]. Both collagen matrices and subepithelial connective tissue graft integration processes are related to a substantial loss of volume during the initial month of healing due to biodegradation. Following this degradation, it takes place a replacement process of the barrier by newly formed connective tissue. Despite this, it is considered that the majority of the matrix is degraded during integration based on the massive volume changes after the grafting procedure. This leads to outcomes comparable to the pre-graft situation [8]. Even more, the generation of more stable collagen matrices via cross-linking with slower biological degradation rate should improve the formation of connective tissue when compared to non-cross-linked ones. Despite this, cross-linking is also involved in triggering foreign body and inflammatory body reactions, which leads to poorer clinical results and wound healing complications [8].

The multiple, variable and available data, when collagen matrices are compared, indicate that differences are attributed not only to the employed collagen matrix, but to a wide array of factors: (i) matrix itself (cross-linked or native collagen), (ii) the experimental clinical scenario (thickening of soft tissue at teeth, in defects of chronic ridge or after guided bone regeneration or immediate implant placement) or the defect model, (iii) the tracing duration (3–10 months), (iv) the placement of the matrix (either epiperiosteal or subperiosteal), (v) folded or unfolded use and matrix thickness, and (vi) histomorphometric methodologies to quantify thickness [8].

## 3. Cellular Therapy for Soft Tissue Augmentation

Recently, bioengineered living cellular therapy has been proposed for soft tissue augmentation and root coverage procedures [19]. In the periodontal wound healing and regeneration field, human living cells cultivation into scaffolding matrices has progressively achieved popularity. Several studies have developed living cellular constructs based on keratinocytes or fibroblasts, either alone or in combination, to be employed as an alternative for autogenous grafts of soft tissue. These constructs have been applied in root coverage and augmentation of keratinized tissue procedures. They pose several advantages, including the reduction of patient morbidity, an endless availability of grafts and good esthetics. A site grafted with a free gingival graft tended to retain palatal tissue characteristics and sites that had received living cellular constructs exhibited significantly higher esthetic results, such as texture and color match, compared to adjacent tissues. The authors have speculated that the better living cellular constructs esthetic results could be attributed to the ability of the material to act as cell-delivery therapy rather than as grafts. This encourages adjacent native cells to migrate into the construct and over it. Native cells stimulation by growth factor and cytokines secretion can, finally, be responsible for a site-appropriate tissue generation. Nevertheless, they finally have argued that constructs made of living cellular components can be the preferred biomaterials, but autogenous soft tissue grafts offer better clinical outcomes in augmentation of keratinized tissue width and root coverage when treating generalized mucosal defects, or when the reduction of patient morbidity is the primary objective.

## 4. Economic and Ethics

Concerning socioeconomic transcendence, specific data to soft tissue injuries are scarce [27], but it has been determined that their economic impact defines them as the major medical problem of the industrialized society [28]. The engineering of tissues aims for the restoration, maintenance or improvement of tissue functions that are considered defective or that have been lost by different pathological conditions, either by the development of biological substitutes or by the reconstruction of tissues. This goal can be achieved by tissue inducing substances delivering (such as growth factors), and by placing cells on or within different matrices [16]. In the tissue engineering market, economic activity has grown considerably in the last few years, due to the appearance of major advances. It has been estimated that the sales of regenerative biomaterials already exceed 240 million of dollars per year [29]. Recently, Seifalin [30] has estimated that the biomaterials market is expected to generate 149.17 billion dollars by 2021.

Despite the excellent cell affinity and biocompatibility, native collagen membranes have several obvious drawbacks for guided tissue regeneration, such as the space-maintaining ability loss in humid conditions, inferior mechanical strength, disease transmission risk to humans for animal-derived collagen, and too-fast biodegradation. These limitations, including the rapid degradation and their poor mechanical properties, have been associated with greater infection susceptibility, abbreviated functional period, and new tissue regeneration [2].

## 5. Conclusions

The patient morbidity associated with a second surgical site when an autogenous soft tissue graft is proposed for soft tissue augmentation represents a drawback. Using human-based tissue derivatives, as an alternative, may be linked to ethical concerns and potential risk of disease transmission. Therefore, the xenogenic collagen matrices proposed for soft tissue augmentation are preferred as they have provided, in general, outstanding clinical outcomes, greater availability, low cost and ability to be harvested in large quantities. However, future strategies should also consider synthetic (alloplastic) matrices for reliable soft tissue augmentation and remodeling strategies.

## Abbreviations

ADM	Acellular dermal matrix
BMP	Bone morphogenetic protein
CTG	Connective tissue graft
ECM	Extracellular matrix
FGF2	Fibroblast growth factor 2
FGG	Free gingival graft
GM-CSF	Granulocyte-macrophage colony-stimulating factor
HAM	Human amniotic membrane
IL	Interleukin
PADM	Porcine-derived acellular dermal matrix
PDGF-BB	Platelet-derived growth factor—two B subunits
TGF-*α*	Transforming growth factor alpha
TGF-*β*	Transforming growth factor beta
TIMP	Tissue inhibitor of metalloproteinase
VEGF	Vascular endothelial growth factor
